# Role for a Web-Based Intervention to Alleviate Distress in People With Newly Diagnosed Testicular Cancer: Mixed Methods Study

**DOI:** 10.2196/39725

**Published:** 2022-10-28

**Authors:** Ciara Conduit, Christina Guo, Allan B Smith, Orlando Rincones, Olivia Baenziger, Benjamin Thomas, Jeremy Goad, Dan Lenaghan, Nathan Lawrentschuk, Lih-Ming Wong, Niall M Corcoran, Margaret Ross, Peter Gibbs, Sophie O'Haire, Angelyn Anton, Elizabeth Liow, Jeremy Lewin, Ben Tran

**Affiliations:** 1 Personalised Oncology Walter and Eliza Hall Institute of Medical Research Parkville Australia; 2 Medical Oncology Peter MacCallum Cancer Centre Melbourne Australia; 3 Sir Peter MacCallum Department of Oncology University of Melbourne Parkville Australia; 4 The Institute of Cancer Research London United Kingdom; 5 The Royal Marsden NHS Foundation Trust London United Kingdom; 6 Ingham Institute for Applied Medical Research & University of New South Wales Liverpool Australia; 7 Department of Surgery Royal Melbourne Hospital University of Melbourne Melbourne Australia; 8 Cancer Surgery Peter MacCallum Cancer Centre Melbourne Australia; 9 Urology St Vincent's Hospital Melbourne Fitzroy Australia; 10 Epworth Healthcare East Melbourne Australia; 11 Victorian Comprehensive Cancer Centre Parkville Australia; 12 Psychosocial Cancer Care and Palliative Care St Vincent's Hospital Melbourne Fitzroy Australia; 13 Cancer Health Services Research Unit Peter MacCallum Cancer Centre Melbourne Australia; 14 Medical Oncology Eastern Health Melbourne Australia; 15 Medical Oncology Monash Health Melbourne Australia; 16 ONTrac at Peter Mac Victorian Adolescence and Young Adult Cancer Service Melbourne Australia

**Keywords:** testicular germ cell tumor, cancer survivors, emotional distress, anxiety disorders, depression

## Abstract

**Background:**

Distress is common immediately after diagnosis of testicular cancer. It has historically been difficult to engage people in care models to alleviate distress because of complex factors, including differential coping strategies and influences of social gender norms. Existing support specifically focuses on long-term survivors of testicular cancer, leaving an unmet need for age-appropriate and sex-sensitized support for individuals with distress shortly after diagnosis.

**Objective:**

We evaluated a web-based intervention, Nuts & Bolts, designed to provide support and alleviate distress after diagnosis of testicular cancer.

**Methods:**

Using a mixed methods design to evaluate the acceptability, feasibility, and impact of Nuts & Bolts on distress, we randomly assigned participants with recently diagnosed testicular cancer (1:1) access to Nuts & Bolts at the time of consent (*early*) or alternatively, 1 week later (day 8; *delayed*). Participants completed serial questionnaires across a 4- to 5-week period to evaluate levels of distress (measured by the National Comprehensive Cancer Network Distress Thermometer [DT]; scored 0-10), anxiety, and depression (Hospital Anxiety and Depression Score [HADS]–Anxiety and HADS-Depression; each scored 0-21). The primary end point was change in distress between consent and day 8. Secondary end points of distress, anxiety, and depression were assessed at defined intervals during follow-up. Optional, semistructured interviews occurring after completion of quantitative assessments were thematically analyzed.

**Results:**

Overall, 39 participants were enrolled in this study. The median time from orchidectomy to study consent was 14.8 (range 3-62) days. Moderate or high levels of distress evaluated using DT were reported in 58% (23/39) of participants at consent and reduced to 13% (5/38) after 1 week of observation. *Early intervention* with Nuts & Bolts did not significantly decrease the mean DT score by day 8 compared with *delayed intervention* (early: 4.56-2.74 vs delayed: 4.47-2.74; *P=*.85), who did not yet have access to the website. A higher baseline DT score was significantly predictive of reduction in DT score during this period (*P*<.001). Median DT, HADS-Anxiety, and HADS-Depression scores reduced between orchidectomy and 3 weeks postoperatively and then remained stable throughout the observation period. Thematic analysis of 16 semistructured interviews revealed 4 key themes, “Nuts & Bolts is a helpful tool,” “Maximizing benefits of the website,” “Whirlwind of diagnosis and readiness for treatment,” and “Primary stressors and worries,” as well as multiple subthemes.

**Conclusions:**

Distress is common following the diagnosis of testicular cancer; however, it decreases over time. Nuts & Bolts was considered useful, acceptable, and relevant by individuals diagnosed with testicular cancer, with strong support for the intervention rendered by thematic analyses of semistructured interviews. The best time to introduce support, such as Nuts & Bolts, is yet to be determined; however, it may be most beneficial as soon as testicular cancer is strongly suspected or diagnosed.

## Introduction

### Background

There have been significant advances in the treatment of testicular cancer in recent decades, such that >97% of individuals can expect a cure [1-3]. While being cancer free, survivors may experience physical, psychological, and social consequences that persist long after their diagnosis and treatment, including cardiovascular morbidity, hypogonadism, second malignancy, and residual chemotherapy toxicities [4-9]. Psychological distress is common immediately following diagnosis [10-13], with a large retrospective study of survivors of testicular cancer suggesting that distress was most significant at this time compared with other periods of their cancer journey [13]. Distress is multifactorial; however, it frequently stems from a perceived lack of information regarding treatment and prognosis [14] and is influenced by risk factors including education level, chronic illness, absence of paid employment, relationship status, and treatment-related factors, such as concomitant use of chemotherapy [6,9,15-18]. Importantly, some individuals may experience persistent symptoms leading to chronic anxiety and depression [19], such that the prevalence and severity of anxiety reported in long-term survivors of testicular cancer is higher than in the general population, with up to 21% of survivors reporting persistent symptoms [9].

Existing support for distress focuses on these long-term survivors, leaving individuals shortly after their diagnosis without adequate resources to support their distress, if required [13,20]. Multiple studies have demonstrated that psychosocial interventions or support help reduce anxiety and depression in people with cancer generally [21-23], and in long-term survivors of testicular cancer [24,25]. However, intervention uptake is variable, particularly in males and young adults [26-29], which may stem from differences in coping strategies and help-seeking behavior and the influence of social gender norms. In addition, there is a lack of age-appropriate and sex-sensitized support for younger people diagnosed with testicular cancer [30,31], further widening this gap and accentuating the need for support to help manage distress proactively and promote long-term psychological health. With this in mind, a pilot study of a web-based psychological intervention in long-term survivors of testicular cancer demonstrated promising acceptability; however, feasibility was limited by poor engagement with the intervention, as evidenced by low module completion rates over time [25]. Where survivors of testicular cancer frequently survive for many decades following curative treatment [32], it is integral to develop novel strategies to adequately address distress at the outset in those who need assistance.

Nuts & Bolts is a web-based intervention funded and operated by the Movember Foundation that could help address this unmet need in patients with recently diagnosed testicular cancer [33]. The intervention comprised the following three domains:

1. Information provision, where individuals can access accurate information about testicular cancer statistics, diagnosis, treatment, and prognosis.
2. “Ask an Expert,” where individuals access responses to frequently asked questions or pose new questions to specialized cancer clinicians and trained peers (with lived experience) and receive personalized responses.
3. “Connect with a Man,” where individuals can access one-on-one peer support from a trained survivor of testicular cancer.

The website requires individuals to self-navigate through the 3 domains according to their specific needs. It was not readily available to the public at the time this study was recruiting; however, the website has since been made available following an official launch.

### Objectives

We undertook a prospective, multicenter, randomized controlled trial to evaluate the acceptability, feasibility, and impact of Nuts & Bolts on distress levels in the weeks following diagnosis of testicular cancer; however, because of poor accrual and anticipated impacts of the COVID-19 pandemic on research personnel, the trial closed early. We then evaluated the prevalence of distress, anxiety, and depression following a recent diagnosis of testicular cancer, changes in symptoms across a period of observation, and an exploration of the lived experience of individuals with newly diagnosed testicular cancer through thematic analysis of semistructured interviews.

## Methods

### Study Design and Participants

This study was designed as a mixed methods, convergent parallel, randomized controlled trial. Eligible participants were aged >18 years, had histologically confirmed testicular cancer within 4 weeks of study consent, were proficient in English and had access to the internet.

Eligible participants were assigned (1:1) to either *early intervention* with access to Nuts & Bolts at the time of study consent or *delayed intervention*, in which access was provided 1 week later. Once access was provided, participants were expected to self-navigate the website according to their specific needs.

### Assessments and Outcomes

**Figure 1:**
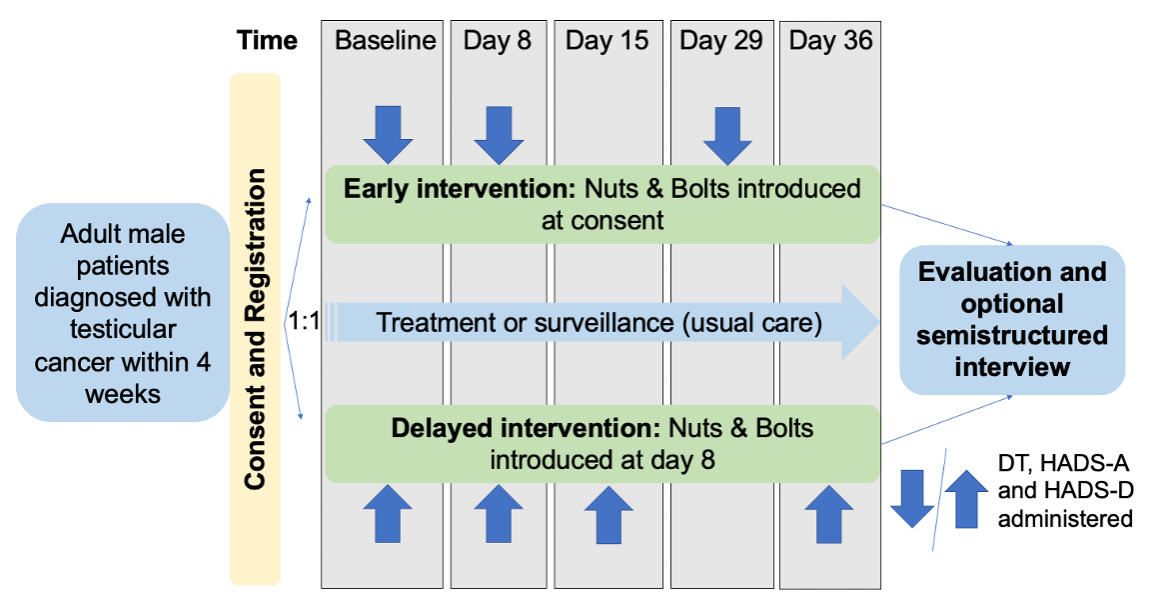
Study scheme. DT: Distress Thermometer; HADS-A: Hospital Anxiety and Depression Scale–Anxiety; HADS-D: Hospital Anxiety and Depression Scale–Depression.

Quantitative data were collected using the National Comprehensive Cancer Network (NCCN) Distress Thermometer (DT) score (0-10) and problem list [34], as well as the Hospital Anxiety and Depression Scale–Anxiety (HADS-A) score (0-21), Hospital Anxiety and Depression Scale-Depression (HADS-D) score (0-21), and Hospital Anxiety and Depression Scale-Total score (0-42) [35]. Participants completed assessments on a web-based portal at study consent and after 1, 2, 4, and 5 weeks, which varied by the assigned study group (Figure 1).

The primary end point was the change in DT score between study consent and day 8 in the *early intervention* group, compared with the control, *delayed intervention* group. Key secondary end points included changes in DT, HADS-A, and HADS-D scores between study consent and day 8 and introduction to Nuts & Bolts and 4 weeks later. Owing to poor accrual, a descriptive analysis of the results obtained from all enrolled participants, regardless of group assignment, was performed. In addition, the acceptability and usability of Nuts & Bolts were evaluated using a supplemental questionnaire delivered after the period of observation.

Qualitative data were collected after completion of the quantitative assessments. Participants were invited to undertake optional, ethically approved semistructured interviews, which were thematically analyzed [36] to explore the lived experiences of individuals following the diagnosis of testicular cancer. Consenting participants were invited to be interviewed using convenience sampling until data saturation was reached. Interviews were undertaken (interviewer was female, registered nurse and research coordinator; see the Acknowledgments section) in accordance with the consolidated criteria for reporting qualitative research recommendations [37]. Telephone interviews lasting 20 to 30 minutes were audio recorded. The interviewer had previous contact with all participants in her role as a research coordinator before the interview; no relevant biases were reported.

### Analyses

We estimated that a sample of 86 participants, allowing for a 20% loss to follow-up, would provide ≥80% power to detect a mean difference of 1.8 between *early* and *delayed intervention* groups when a change in DT scores from baseline to day 8 were assessed using analysis of covariance where study arm and baseline DT score treated as covariates. In addition, linear regression was performed to explore the impact of the study arm and baseline DT score on the reduction in DT. Other quantitative data were analyzed using simple descriptive statistics. Odds ratios (ORs) were used to explore associations among categorical data, and the log method was used to calculate 95% CIs. For comparisons of mean scores between time points and subgroups, paired or independent 2-tailed *t* tests were used and are further outlined in the Results section. Statistical significance was defined as a 2-tailed *P* value of ≤.05. All statistical analyses were performed using Stata (version 14.2; IBM Corp) and Microsoft Excel for Mac (version 16.5).

Qualitative semistructured interview data were thematically analyzed, systematically identifying, organizing, and providing insight into patterns of meaning (themes) across the data set [36]. Interview transcripts were independently coded using NVivo (version 10; QSR International) by a member of the research team naïve to intervention allocation. A subset of the interviews (n=3) was coded by a second researcher to ensure concordance, and differences in coding were resolved through discussion. The themes were derived from the data. A constant comparative method involving moving back and forth between the interview transcripts, coded data extracts, and themes generated was used to ensure that the thematic hierarchy accurately reflected the interview data. Subthemes and themes were finalized in discussions with a second researcher to reduce the risk of individual biases affecting the results. The participants did not provide feedback on the findings.

Study data were collected and managed using REDCap (Research Electronic Data Capture; Vanderbilt University) [38,39], electronic data capture tools hosted by the Clinical Translation Centre, Walter and Eliza Hall Institute of Medical Research.

### Ethical Considerations

This study was approved by the Melbourne Health Human Research Ethics Committee (MH-2018-157301). Local ethical and governance approval was obtained from all participating sites. All participants provided written informed consent based on the Declaration of Helsinki principles [40] and were not financially compensated for their involvement in the study. All data collected during the study were deidentified; participants were provided with a unique identification code at the time of registration.

## Results

### Participants

**Figure 2:**
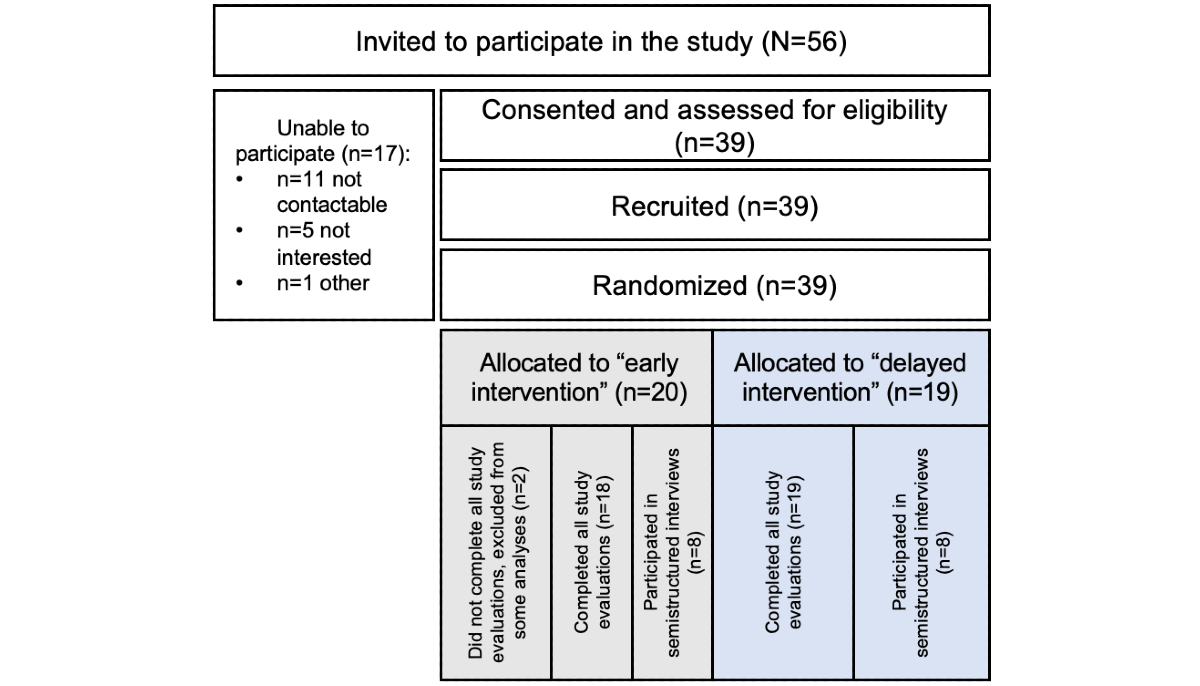
CONSORT (Consolidated Standards of Reporting Trials) diagram.

Between April 2019 and April 2020, of the 56 invited participants, 39 (70%) participants from 4 sites consented to the trial and were randomly assigned to *early intervention* (20/39, 51%) or *delayed intervention* (19/39, 49%; Figure 2). The accrual was closed early because of the anticipated impact of the COVID-19 pandemic on research personnel and hospital resources at coordinating and recruiting centers. Overall, 119 study questionnaires were completed during the observation period, and 95% (37/39) of the participants completed all study assessments. The median participant age was 32 (range 24-55) years, and the median time from orchidectomy was 14.8 (range 3-62) days at study consent (Table 1). In all, 5% (2/39) of the participants were enrolled >4 weeks after the diagnosis of testicular cancer (enrollment violation).

**Table 1:** Baseline characteristics (N=39).

				Eligible participants (n=39)
Age at consent (years), median (range)				32.4 (24-55)^a^
**TNM^b^ stage, n (%)**				
	I			32 (82)
	II-III			4 (10)
	Not stated			3 (8)
**Ethnicity, n (%)**				
	White			34 (87)
	Asian			4 (10)
	Other			1 (3)
**Relationship status, n (%)**				
	Single			12 (31)
	Married or de facto			21 (54)
	In a relationship			6 (15)
**Highest level of education completed, n (%)**				
	High school			5 (13)
	Apprenticeship			4 (10)
	Tertiary			30 (77)
Paid employment, n (%)				36 (92)
**Mental health history, n (%)**				
	Previous history of mental ill health			8 (21)
	Currently receiving mental health support			9 (23)
**Orchidectomy performed, n (%)**				
	Yes			36 (92)
	Not stated			3 (8)
Time from orchidectomy (days), median (range)				14.8 (3-62)
Medical oncologist involvement at time of enrollment, n (%)				24 (62)
**Planned treatment, n (%)**				
	Surveillance			30 (77)
	Chemotherapy			5 (13)
	Not stated			4 (10)
**Baseline level of distress**				
	**DT^c^**			
		Median score: all participants, median (range)	5 (0-8)	
		Mean score: consented <14 days since orchidectomy, mean (range)	5.2 (1-8)	
		Mean score: consented >14 days since orchidectomy, mean (range)	3.7 (0-7)	
	**HADS-A^d^**			
		Median score: all participants, median (range)	5 (0-15)	
		Mean score: consented <14 days since orchidectomy, mean (range)	6.9 (2-15)	
		Mean score: consented 14 days since orchidectomy, mean (range)	4.4 (0-11)	
	**HADS-D^e^**			
		Median score: all participants, median (range)	3 (0-10)	
		Mean score: consented <14 days since orchidectomy, mean (range)	4.6 (0-9)	
		Mean score: consented >14 days since orchidectomy, mean (range)	3.1 (0-10)	
	**Moderate or high levels of distress, n (%)**			
		DT≥5	23 (59)	
		HADS-T^f^≥11	15 (38)	

^a^Excluding 2 participants in whose date of birth was incorrectly recorded.

^b^TNM: tumor, node, metastases.

^c^DT: Distress Thermometer.

^d^HADS-A: Hospital Anxiety and Depression Scale–Anxiety.

^e^HADS-D: Hospital Anxiety and Depression Scale–Depression.

^f^HADS-T: Hospital Anxiety and Depression Scale–Total.

### Quantitative Results

#### Baseline Characteristics

Distress was reported by most participants at baseline on DT. The median DT score was 5 (range 0-8), with 53% (21/39) of all participants reporting moderate (DT score≥5 and <8) and 5% (2/39) reporting high-level (DT score≥8) distress. Baseline DT scores were not associated with key demographic risk factors for distress, including preexisting mental health history (moderate distress: OR 2.5, 95% CI 0.4-14.2), lower level of education (moderate distress: OR 0.8, 95% CI 0.3-3.9), or relationship status (moderate distress: OR 1.0, 95% CI 0.3-4.1) in our study (Table S1 in Multimedia Appendix 1). However, participants consenting to Nuts & Bolts <14 days following orchidectomy reported higher DT (5.2 vs 3.7; *P*=.04) and HADS-A (6.9 vs 4.4; *P*=.03) scores than participants who consented >14 days following orchidectomy in 2-tailed *t* test analyses.

Emotional and physical problems dominated the NCCN problem list at baseline, with nervousness, worry, fear, sadness, fatigue, feeling swollen, and pain reported by at least half of the participants (at least 22/39, 56%; Table S2 in Multimedia Appendix 1).

#### Difference Between *Early* and *Delayed* Intervention Groups Between Consent and Day 8

Early intervention with Nuts & Bolts did not significantly reduce mean DT scores on day 8 compared with those for delayed intervention after adjusting for baseline DT score (*P=*.85) when analyzed using an analysis of covariance (Tables S3 and S4 in Multimedia Appendix 1). The primary endpoint was not achieved. Using linear regression analysis, a higher baseline DT score was associated with a statistically significant reduction in DT across this period (*P*<.001) for all participants, but study arm was not.

#### Change in Distress, Anxiety, and Depression During Follow-up for the Whole Cohort

When analyzed as a whole, regardless of the group assignment, levels of distress evaluated using DT significantly declined between baseline evaluation and after 1 week in a paired 2-tailed *t* test analysis (4.6 vs 2.7; *P*<.001; Table S3 in Multimedia Appendix 1).

In contrast to the baseline evaluation, only 13% (5/38) of the participants reported moderate distress on DT after 1 week of observation, and none of the participants reported high levels of distress. Levels of anxiety evaluated using HADS-A did not change between baseline and 1-week later (5.7 vs 5.1; *P=*.26); however, depression scores reduced significantly across the same period (3.8 vs 3.1; *P*=.04; Table S4 in Multimedia Appendix 1).

When analyzed by time from orchidectomy rather than time from study entry, median DT, HADS-A, and HADS-D scores reduced most between 1 and 4 weeks following orchidectomy and then remained largely stable throughout the remainder of the observation period (Table 2).

**Table 2:** Median levels of distress, anxiety, and distress during observation.

Weeks following orchidectomy	Number of observations	DT^a^ score, median (range)	HADS-A^b^ score, median (range)	HADS-D^c^ score, median (range)
≤1	10	5 (1-8)	6.5 (2-10)	4.5 (0-9)
>1 to ≤2	14	4.5 (1-8)	6.5 (0-15)	4.5 (1-9)
>2 to ≤3	20	2 (0-6)	4 (0-10)	2 (0-7)
>3 to ≤4	19	3 (0-7)	6 (2-11)	2 (0-10)
>4 to ≤5	24	2 (0-7)	5 (0-12)	2 (0-9)
>5 to ≤6	11	2 (0-5)	4 (0-11)	2 (0-10)
>6	21	1 (0-5)	4 (0-11)	2 (0-14)

^a^DT: Distress Thermometer.

^b^HADS-A: Hospital Anxiety and Depression Scale–Anxiety.

^c^HADS-D: Hospital Anxiety and Depression Scale-Depression.

#### Evaluation of Acceptability and Feasibility

Overall, 95% (37/39) of the participants completed the evaluation of acceptability and feasibility at the conclusion of the study. Most participants expressed that Nuts & Bolts was easy to use (37/37, 100%), relevant (36/37, 97%), and useful (31/37, 84%). Almost two-thirds (24/37, 65%) used the “Ask an Expert” module, with 87% (20/24) of responders agreeing that this module was useful, although some noted that their questions were not answered. A smaller proportion used the “Connect with a Man” module (5/37, 14%) and all agreed that this module was useful (5/5, 100%; Table 3). Participants who did not use “Connect with a Man” reported that other Nuts & Bolts domains and resources, such as family or friends, reduced the potential utility of this module. Many offered support for the idea of “Connect with a Man,” and some enrolled to become trained peers following the study (Table 3).

**Table 3:** Responses to poststudy questionnaire of acceptability and feasibility.

Statement asked	Response					
	Strongly disagree, n (%)	Disagree, n (%)	Unsure, n (%)	Agree, n (%)	Strongly agree, n (%)	Respondents, n (% of users)
Nuts & Bolts was useful to me	0 (0)	3 (8)	3 (8)	13 (35)	18 (49)	37 (95)
Nuts & Bolts was relevant to me	0 (0)	0 (0)	1 (3)	24 (65)	12 (32)	37 (95)
Nuts & Bolts was easy to use	0 (0)	0 (0)	0 (0)	11 (30)	26 (70)	37 (95)
I could understand the information provided by Nuts & Bolts	0 (0)	0 (0)	0 (0)	10 (27)	27 (73)	37 (95)
The length of the content on Nuts & Bolts was appropriate to me	0 (0)	1 (3)	3 (8)	9 (25)	23 (64)	36 (92)
I found the “Ask an Expert” section useful	0 (0)	1 (4)	2 (9)	14 (61)	6 (26)	23 (96)
I found the “Connect with a Man” section useful	0 (0)	0 (0)	0 (0)	2 (40)	3 (60)	5 (100)

### Thematic Analysis

#### Overview

Over three-fourths of the participants (30/39, 77%) provided consent to participate in the optional, semistructured interviews, and using convenience sampling, 16 interviews were conducted. This group was representative of the studied population with a median age of 30.5 (range 24.1-54.5) years, and 50% (8/16) were assigned to each study group. Most participants were White (14/16, 88%), married or in a de facto relationship (11/16, 69%), and diagnosed with stage I testicular cancer (10/16, 63%; data not shown).

**Figure 3:**
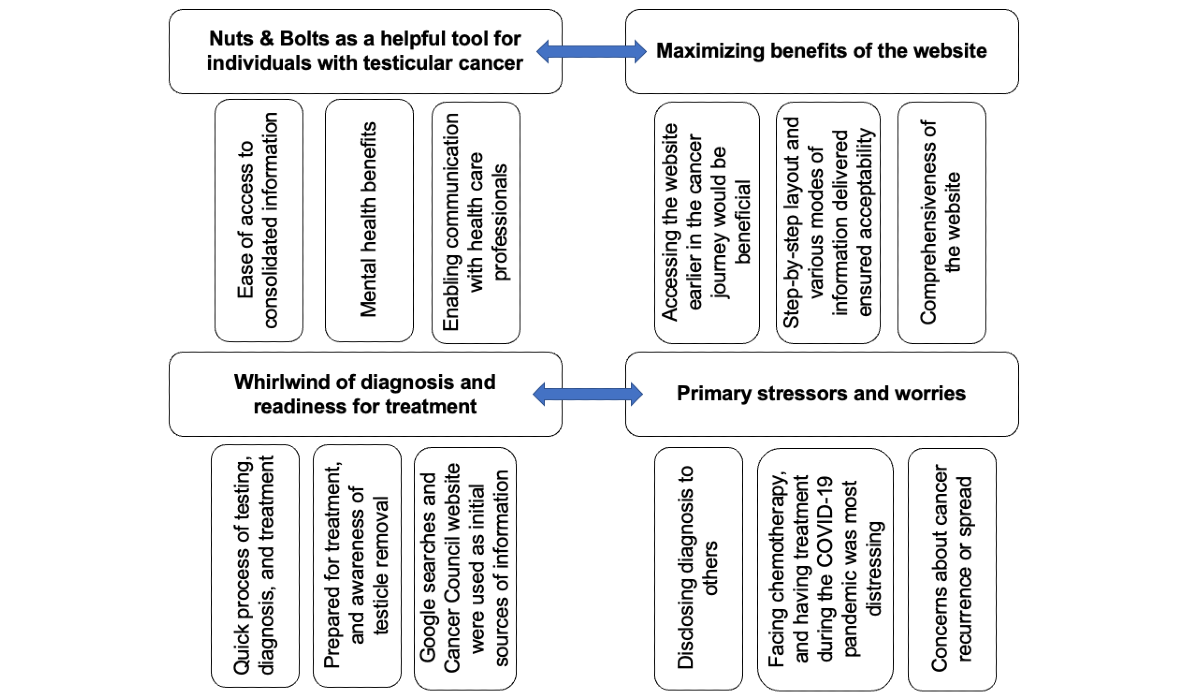
Thematic map.

Thematic analysis of interviews generated 4 main themes regarding participants’ experiences following the diagnosis of testicular cancer and use of Nuts & Bolts (Figure 3). Additional illustrative quotes related to the subthemes are shown in Table 4.

**Table 4:** Themes, subthemes, and illustrative quotes from thematic analyses.

Theme and subthemes		Illustrative quotes
**1. Nuts & Bolts website as a helpful tool**		
	A. Ease of access to consolidated information	“The most helpful thing about the site is the fact that it consolidates the information that you’re after for this specific condition and that’s something that’s not readily available.” [Tertiary educated, in a relationship, aged 55 years]
	B. Mental health benefits	“And I think...[the website] was good because when I was about to start to get a bit anxious so that like ‘Oh, God, what if, what if, what ifs,’ I could read the information to just reassure myself, I guess, with the general facts...” [Tertiary educated, in a relationship, aged 34 years]
	C. Enabling communication with health care professionals	“...[The website] gave me the proper questions that I need to ask, not only the oncologist, but also the nurses when I went into chemo.” [High school educated, in a relationship, aged 49 years]
**2. Maximizing the benefits of the website**		
	A. Accessing the website earlier in the testicular cancer journey would be beneficial	“I reckon...[the website] would be most useful pretty much as soon as you get diagnosed...Look, I would suggest probably once you’ve had some tests done with your GP, if it’s made available, then I think that would be beneficial...before going to see a urologist...” [Tertiary educated, single, aged 30 years]
	B. Step-by-step layout and varied mode of information delivery ensured acceptability	“...[the website] has a step by step, and it explains each stage, and then you can drill down on the information...and then it explains as you go through the journey...And I think...[the website] is kind of like, ‘Here, we’ll help you’—like just a path. It just lays out the path that you need to go along.” [Tertiary educated, single, aged 33 years]; “I’d say definitely those videos, it just sort of put a human touch on the whole situation...” [High school educated, in a relationship, aged 30 years]
	C. Comprehensiveness of the website	“I think everything is pretty good. I didn’t feel like anything was lacking.” [Tertiary educated, in a relationship, aged 30 years; “delayed intervention”]; “...some mental health support would have been good on the website...even if it was just like maybe a link or something like that to like—a support group...a psychologist or who to talk to...” [Tertiary educated, in a relationship, aged 33 years]
**3. Whirlwind of diagnosis and readiness for treatment**		
	A. Quick process of testing, diagnosis, and treatment	“Yeah, it was quick. I didn’t expect it to be so soon, like it’s good that it was. So I saw her [his GP] on a Monday and then the Wednesday, it was the surgery booking...” [Tertiary educated, in a relationship, aged 30 years]
	B. Prepared for treatment and awareness of testicle removal	“...once I found out they were gonna need to remove it, you sort of don’t really care. So, all that aesthetic stuff that normally comes with being a bloke, when you find out you have something like that in your body, you don’t really care. You just wanna get rid of it.” [High school educated, male, in a relationship, aged 30 years]; “I knew it had to be removed and, I suppose, I didn’t feel great about it.” [High school educated, in a relationship, aged 49 years]
	C. Google searches and Cancer Council website were used as initial sources of information	“...both my partner and I did a bit of reading online. We didn’t do a lot because we didn’t wanna...scare ourselves...I had a brief read of the Cancer Council site, which was probably the most informative that I came across. And I sort of—after I got the general gist, I went, ‘Yep, all right, that’s enough.’” [High school educated, in a relationship, aged 30 years]
**4. Primary stressors and worries**		
	A. Disclosing the diagnosis to others	“I found it really hard trying to control the people around me. I found that the biggest stress for me because they would hear the word cancer and kind of freak out a little bit.” [Tertiary educated, male, in a relationship, unknown age]
	B. Facing chemotherapy and having treatment during the COVID-19 pandemic was most distressing	“I was quite worried about that and how my body was gonna handle [the chemotherapy].” [High school educated, in a relationship, aged 49 years]; “Honestly with the COVID-19 scenario at the moment, it’s a little bit hard to me as well. My parents are stuck over in Western Australia, so they can’t actually come here. So I’m, unfortunately, living on my own at the moment, so I’m having to look after myself a bit which is a little bit distressing but, look, you have to acclimatise and it is what it is.” [Tertiary educated, male, single, receiving chemotherapy, aged 30 years]
	C. Concerns about cancer recurrence or spread	“I’ve got positive results and it looked good, there’s no point living in fear, I suppose, or whether it affects your life...But with that said, I do have a level of fear that it will come back, or it will manifest somewhere else.” [Tertiary educated, in a relationship, aged 38 years]

#### Nuts & Bolts Is a Helpful Tool

Nuts & Bolts was considered valuable throughout the journey with testicular cancer, including in participants with recurrent testicular cancer and those with high health literacy:

I think [Nuts & Bolts is useful at] every stage of the journey to be honest. Right from being given diagnosis through to any potential surgery and then post-surgery and the chemo and even recovery.

High school educated, in a relationship, aged 49 years

A total of 3 main subthemes were identified in this study. Participants reported that Nuts & Bolts provided consolidated access to reliable information (subtheme 1A). Specifically, many liked how the webpage explained what to expect for different disease stages (localized and advanced) and the role of various team members in their care. In addition, participants described feeling less anxious and distressed after accessing the resource (subtheme 1B), as the information provided offered them realistic expectations about treatment and prognosis. Some participants reported that Nuts & Bolts also helped lessen the fear of cancer recurrence and improved their baseline knowledge of testicular cancer, which made it easier to ask questions and communicate with their health care team (subtheme 1C).

#### Maximizing the Benefits of the Website

All participants accessed Nuts & Bolts after study enrollment, with timing dependent on their group assignments. Most individuals had already commenced treatment, most commonly orchidectomy, when they first accessed the website:

...*I discovered [Nuts & Bolts] sort of after my operation. So, the worst of it had sort of been over.* [High school educated, in a relationship, aged 30 years]

A total of 3 main subthemes were identified. Some participants assigned to either intervention groups perceived the timing of introduction to Nuts & Bolts as “late” with consensus that the optimal timing would be before seeing a urologist and orchidectomy (subtheme 2A). Despite this, participants considered Nuts & Bolts valuable and acknowledged the logistical barriers to providing earlier access because of the rapidity of diagnosis and treatment. The clear, step-by-step layout and varied mode of information delivery through images, videos, and patient testimonials was also highly acceptable (subtheme 2B). Nuts & Bolts was generally considered comprehensive (subtheme 2C); however, some participants suggested that additional information about the recovery time after treatment, chemotherapy, testicular cancer subtypes, and mental health support would be helpful.

#### “Whirlwind” of Diagnosis and Treatment Readiness

A total of 3 main subthemes were identified. Most participants perceived the diagnosis process and commencing treatment to be rapid (subtheme 3A), with some expressing “disbelief” or “shock” following their diagnosis. However, this rapid pace was valued by other participants, who were keen to *get rid of it* [tertiary educated, single, aged 30 years]. Only 1 participant indicated that they would have preferred more time to process the available information (subtheme 3B). In addition, most participants were aware that they would require orchidectomy and indicated that they felt prepared for this procedure. Some participants noted that they were not worried about their testicles being removed, while others felt *“devastated.”*

Before study enrollment, Google searches and government-endorsed websites such as the Australian “Cancer Council” were commonly used to seek information (subtheme 3C). However, a few participants were hesitant because of concerns about negative anecdotes and information quality. Some participants accessed information from family, friends, or other physicians, others did not actively seek information before their diagnosis, citing a preference not to be overwhelmed by information. A participant voiced that they would seek out Nuts & Bolts as their first resource if they experienced recurrence or contralateral testicular cancer, obviating the need for broad Google searches.

#### Primary Stressors and Worries

The participants expressed that various emotions and stressors arose following their diagnosis of testicular cancer. The most distressing concerns were related to social impact following diagnosis and treatment-related concerns.

A total of 3 main subthemes were identified. Many participants described communicating information to friends and family and concerns about managing their emotional reactions as a significant source of distress (subtheme 4A). Some participants reported that investigations, particularly scans and chemotherapy treatments, added additional sources of stress during their journey. For participants enrolled in 2020, the impact of the COVID-19 pandemic and risk associated with attending hospitals for treatment during this period added further complexity to their experience (subtheme 4B). Finally, many participants reported fear of cancer recurrence or spread; however, this did not appear to cause sustained distress or functional impairment in most cases (subtheme 4C). Several participants indicated they were explicitly maintaining a “positive attitude” and avoiding thoughts about recurrence. Other concerns raised by the participants included the risk of infertility and contralateral testicular cancer in the future.

## Discussion

### Principal Findings

Distress identification is vital when caring for patients diagnosed with cancer [19]. Our study of individuals with recently diagnosed testicular cancer found that more than half of the participants reported moderate distress at the time of study enrollment, with the highest distress observed in participants within 14 days of orchidectomy, as in previous research [10-13]. Reassuringly, distress levels decreased over time, with a significant change in mean DT scores seen throughout the course of observation and as time increased from diagnosis, with only 13% (5/38) of the participants reporting moderate distress after 1 week of observation (approximately 3 weeks after orchidectomy). Notably, depressive symptoms were less common than anxiety in our cohort, which mirrors existing research [6,41].

Although the primary outcome of this study was not met and earlier introduction to Nuts & Bolts did not lead to a significant reduction in distress on day 8, thematic analysis of semistructured interviews occurring after completion of quantitative assessments emphasized a high level of perceived utility for Nuts & Bolts. Multiple participants indicated a strong preference for access to Nuts & Bolts at the time of diagnosis, when their distress was highest, while acknowledging its usefulness during and after treatment. Importantly, the introduction of Nuts & Bolts did not negatively affect distress, and thematic analysis and poststudy evaluations strongly endorse its ongoing role in supporting individuals following the diagnosis of testicular cancer. Partnerships between researchers and nongovernment and industry organizations are key to the sustained dissemination of web-based interventions in cancer care [42]. As this study was completed, Movember has formally launched and promoted the availability of Nuts & Bolts.

Making Nuts & Bolts available to individuals earlier in the process of diagnosing and treating testicular cancer may increase its clinical utility. The perceived “late” introduction to Nuts & Bolts may have lessened its clinical utility. A preference for earlier intervention, that is, before orchidectomy, was highlighted by participants in semistructured interviews and may be appropriate to help ameliorate the significant distress and whirlwind of diagnosis they experience in some individuals where a testicular cancer diagnosis is *strongly* suspected based on preoperative information. Other studies evaluating psychological interventions for the management of distress in patients with cancer, have similarly highlighted that earlier interventions lead to reduced stress, improved quality of life, and superior clinical outcomes [18]. With conflicting reports regarding the prevalence of long-term distress in survivors of testicular cancer [9,10,43], early intervention is important for those who wish to receive it. In addition, as a clinical trial requiring consent, the study may have introduced a potential selection bias for “active copers” rather than individuals with passive coping strategies [6], which may be reflected in the 70% (39/56) response rate for study involvement. These individuals are likely to seek additional information following their diagnosis as opposed to individuals with passive coping strategies. Sociodemographic information of nonconsenting individuals was not collected.

The potential sources of distress elicited from participants were wide ranging, with domains of emotional problems, such as nervousness, worry, fear and sadness and physical problems, such as pain, fatigue and “feeling swollen” dominating the NCCN problem list tool at study entry. Notably, these stressors reduced over time, with a comparatively small number of participants reporting these problems after 4 weeks of observation. This may relate to the resolution of postoperative symptoms, particularly pain and “feeling swollen,” and adjustment to the new diagnosis over time. In addition, thematic analysis revealed important concerns regarding communicating with family and friends, fear of cancer recurrence or spread, potential toxicity from chemotherapy, and risks posed by the COVID-19 pandemic while undergoing treatment. Although only raised by a small number of participants in the semistructured interviews in our study, previous research has identified significant concerns about fertility and sexual health following a cancer diagnosis [44-47], which is relevant to survivors of testicular cancer. Regardless, Nuts & Bolts was able to address multiple sources of distress for some participants by providing accurate information about their diagnosis, treatment options, and prognosis, while others relied on their health care team, alternate resources, and family or friends to fill these gaps.

Overall, Nuts & Bolts was considered relevant, user-friendly, and acceptable by most participants. These findings are consistent with previous studies, which reported high levels of patient satisfaction with web-based and mobile-based psychosocial interventions [48-55], particularly those encouraging patient empowerment [56], such as Nuts & Bolts. This is significant for survivors of testicular cancer, given the barriers to engagement that this unique cohort faces over and above other populations with cancer [26-31]. As almost all individuals recruited in our study reported ethnicity, individuals from culturally and linguistically diverse backgrounds may require alternate support geared toward their needs in the future.

### Strengths and Limitations

Our study had several strengths, including its prospective design with limited missing data and the inclusion of a mixed methods analysis derived from questionnaires and thematic analysis of semistructured interviews highlighting key issues for survivors after diagnosis. Unfortunately, owing to poor accrual and anticipated impacts of the COVID-19 pandemic on hospital resources and recruiting and coordinating centers, the study was closed early, and consequently, the primary end point was underpowered, and we were unable to draw firm conclusions about the differential impact of Nuts & Bolts on distress after 1 week. The instruments that have been validated in multiple clinical settings may also have been too crude to adequately evaluate changes over a short period, which may have also limited the interpretation [57].

In addition, our primary end point may have been inadvertently hampered by the study design. When designing the study, we felt that withholding access to a potentially valuable clinical resource from patients in the *delayed* group for more than 1 week was unethical. Although the baseline characteristics were balanced across both groups, our results showed that distress was highest in participants who were enrolled within 14 days of orchidectomy, and the baseline level of distress was the only covariate associated with a significant decline in DT score after 1 week of observation. Therefore, a 1-week delay in the introduction of Nuts & Bolts from the time of study consent was unlikely to have a significant impact on distress. Instead, prospectively enrolling participants before orchidectomy to ensure that questionnaires were completed at uniform periods postoperatively may have overcome this; however, this approach risked missing potential participants owing to the rapidity of diagnostic workup and delays in referral for the trial. Despite this limitation, the thematic analysis of semistructured interviews and observation over time of individuals’ distress following a recent testicular diagnosis adds valuable data to the literature and remains a significant strength.

### Conclusions

High levels of distress are observed following a diagnosis of testicular cancer; however, this decreases over time. Nuts & Bolts is an acceptable and feasible tool to help address distress in individuals recently diagnosed with testicular cancer, empowering them to seek information relating to their diagnosis and potentially improve preparedness for treatment using a model appropriate for its target population. The optimal timing of introduction remains unclear; however, early access to appropriate support appears to be key to maximizing benefit and ameliorating the whirlwind associated with diagnosis and treatment. On the basis of these outcomes, the intervention was rolled out in a broader community of individuals diagnosed with testicular cancer.

## Appendix

Multimedia Appendix 1 Baseline characteristics (N=39).

## Abbreviations

DT: Distress Thermometer
HADS-A: Hospital Anxiety and Depression Scale–Anxiety
HADS-D: Hospital Anxiety and Depression Scale-Depression
NCCN: National Comprehensive Cancer Network
OR: odds ratio
REDCap: Research Electronic Data Capture
