# Supplementary material for: Role for a Web-Based Intervention to Alleviate Distress in People With Newly Diagnosed Testicular Cancer: Mixed Methods Study
Source: JMIR Cancer. 2022 Oct 28;8(4):e39725. doi: 10.2196/39725 (PMC9652729; doi:10.2196/39725)
Supplement: Multimedia Appendix 1 [file cancer_v8i4e39725_app1.docx]

Supplemental Table I: Impact of key demographic factors on presence of moderate- or high-level distress at baseline

| **Demographic factor** | | **Median DT score (range)** | **Moderate or high distress (DT), n (%)** | **OR (95% CI)** |
| --- | --- | --- | --- | --- |
| Highest level of education | Tertiary | 5 (1-8) | 18 (60) | 1.2 (0.3-5.4) |
|  | Apprenticeship or High school | 5 (0-7) | 5 (33) |  |
| Relationship status | Married/de facto/in a relationship | 5 (0-8) | 16 (67) | 1 (0.3-4.1) |
|  | Single | 5 (1-7) | 7 (58) |  |
| Paid employment | Yes | 5 (0-8) | 20 (56) | ∞ |
|  | No | 7 (6-8) | 3 (100) |  |
| Pre-existing mental ill health | Yes | 5 (3-8) | 6 (75) | 2.5 (0.4-14.2) |
|  | No | 5 (0-8) | 17 (55) |  |
| Treatment plan | Chemotherapy | 5 (1-7) | 3 (60) | 1.3 (0.2-9) |
|  | Surveillance | 5 (0-8) | 16 (53) |  |
| Met with medical oncologist | Yes | 5 (0-8) | 14 (58) | 0.9 (0.3-3.5) |
|  | No | 5 (1-8) | 9 (60) |  |
| Disease stage (TNM) | 1 | 5 (0-8) | 17 (53) | 0.4 (0.04-4) |
|  | 2 or 3 | 5 (4-7) | 3 (75) |  |

Legend: DT = Distress Thermometer; OR = odds ratio, 95% CI = 95% confidence interval

Supplemental Table II: NCCN Problem List

| **Domain/Problem** | **Study consent, n (%) (n=39)** | **4-weeks post-intervention, n (%) (n=37)** |
| --- | --- | --- |
| *Practical problems* | | |
| Child care | 2 (5) | 1 (3) |
| Housing | 1 (3) | 1 (3) |
| Insurance/Financial | 4 (10) | 2 (5) |
| Transportation | 2 (5) | 2 (5) |
| Work/School | 4 (10) | 1 (3) |
| Treatment Decisions | 3 (8) | 1 (3) |
| *Family problems* | | |
| Dealing with children | 1 (3) | 0 (0) |
| Dealing with partner | 5 (13) | 0 (0) |
| Ability to have children | 3 (8) | 3 (8) |
| Family health issues | 7 (18) | 1 (3) |
| *Emotional Problems* | | |
| Depression | 7 (18) | 2 (5) |
| Fears | 26 (67) | 3 (8) |
| Nervousness | 32 (82) | 2 (5) |
| Sadness | 22 (56) | 4 (11) |
| Worry | 31 (79) | 7 (19) |
| Loss of interest in usual activities | 10 (26) | 2 (5) |
| *Spiritual/religious concerns* | 0 (0) | 0 (0) |
| *Physical problems* | | |
| Appearance | 7 (18) | 0 (0) |
| Bathing/dressing | 12 (31) | 0 (0) |
| Breathing | 1 (3) | 0 (0) |
| Changes in urination | 2 (5) | 0 (0) |
| Constipation | 8 (21) | 0 (0) |
| Diarrhoea | 0 (0) | 0 (0) |
| Eating | 1 (3) | 0 (0) |
| Fatigue | 22 (56) | 1 (3) |
| Feeling swollen | 22 (56) | 0 (0) |
| Fevers | 1 (3) | 0 (0) |
| Getting around | 13 (33) | 0 (0) |
| Indigestion | 2 (5) | 0 (0) |
| Memory/concentration | 7 (18) | 0 (0) |
| Mouth sores | 1 (3) | 0 (0) |
| Nausea | 1 (3) | 0 (0) |
| Nose dry/congested | 4 (10) | 0 (0) |
| Pain | 27 (69) | 0 (0) |
| Sexual | 7 (18) | 0 (0) |
| Skin dry/itchy | 9 (23) | 0 (0) |
| Sleep | 12 (31) | 1 (3) |
| Substance use | 0 (0) | 0 (0) |
| Tingling in hands/feet | 0 (0) | 0 (0) |

Supplemental Table III: Mean Levels of Distress, Anxiety and Distress at Key Time Points

| **Time point** | **Baseline, mean score (standard deviation)** | | | | **Day 8, mean score (standard deviation)** | | | | **Day 15, mean score (standard deviation)** | | | | **4-weeks post-intervention, mean score (standard deviation)**^a^ | | | |
| --- | --- | --- | --- | --- | --- | --- | --- | --- | --- | --- | --- | --- | --- | --- | --- | --- |
| **Tool** | *n* | *DT* | *HADS-A* | *HADS-D* | *n* | *DT* | *HADS-A* | *HADS-D* | *n* | *DT* | *HADS-A* | *HADS-D* | *n* | *DT* | *HADS-A* | *HADS-D* |
| *Early* | 20 | 4.5  (2) | 5.5  (3.1) | 3.9  (2.5) | 19 | 2.7  (1.3) | 5  (3.0) | 3.2  (2.4) | NA | | | | 18 | 2.8  (1.8) | 4.8  (2.6) | 3.5  (3.5) |
| *Delayed* | 19 | 4.5  (2.4) | 5.5  (3.9) | 3.6  (3.2) | 19 | 2.7  (1.6) | 5.2  (3.2) | 3  (3) | 19 | 2  (2) | 4.3  (3.4) | 2.9  (6.23) | 19 | 1.6  (1.8) | 3.9  (3.1) | 2.6  (3.2) |
| *All* | 39 | 4.5 (2.2) | 5.5 (3.4) | 3.6 (3.2) | 38 | 2.7 (1.4) | 5.1 (3.1) | 3.1 (2.7) | NA | | | | 37 | 2.2 (1.9) | 4.3 (2.9) | 3 (3.3) |

Legend: DT= distress thermometer, HADS-A= Hospital Anxiety and Depression Scale Anxiety Score, HADS-D= HADS-Depression Score

^a^ assessed at Day 29 in participants assigned to ‘early intervention’ and Day 36 in participants assigned to ‘delayed intervention’

Supplemental Table IV: Key Comparisons of Levels of Distress During Study

| **Analysis** | | **Tool** | | |
| --- | --- | --- | --- | --- |
|  |  | **DT** | **HADS-A** | **HADS-D** |
|  |  | *P* value | | |
| *Early* | Baseline *versus* Day 8^a^ | *<.001* | .27 | .15 |
|  | Baseline *versus* Day 29^a^ | *<.001* | .22 | .63 |
| *Delayed* | Baseline *versus* Day 8^a^ | *.01* | .65 | .14 |
|  | Baseline *versus* Day 36^a^ | *<.001* | *.04* | *.04* |
|  | Day 8 *versus* Day 36^a^ | *.01* | .*03* | .43 |
| Early *versus Delayed* | Baseline^b^ | .97 | .98 | .73 |
|  | Day 8^b^ | 1.00 | .84 | .81 |
|  | *Change* from Baseline to Day 8^c^ | .85 | .67 | .56 |
| *Grouped* | Baseline *versus* Day 8 | *<.001* | .26 | .*04* |
|  | Introduction to Nuts & Bolts *versus* 4 weeks later^a^ | *<.001* | *.01* | .39 |

Legend: DT= distress thermometer, HADS-A= Hospital Anxiety and Depression Scale Anxiety Score, HADS-D= HADS-Depression Score

^a^ calculated using paired t-test, ^b^ calculated using independent t-test, ^c^ calculated using ANCOVA after adjusting for baseline score
